# Supplementary material for: Associations of the intestinal microbiota with plasma bile acids and inflammation markers in Crohn’s disease and ulcerative colitis
Source: Sci Rep. 2025 Oct 8;15:35039. doi: 10.1038/s41598-025-18106-7 (PMC12508173; doi:10.1038/s41598-025-18106-7)
Supplement: Supplementary file 4 — Supplementary Information 4. [file 41598_2025_18106_MOESM4_ESM.docx]

Supplementary material:

Supplementary table 1: Taxonomic profiles of all samples included

Supplementary table 2: Bile acid profiles of all samples included

Supplementary table 3: Olink inflammation profiles of all samples included
